# Ultrasensitive Biosensor for the Detection of *Vibrio cholerae* DNA with Polystyrene-co-acrylic Acid Composite Nanospheres

**DOI:** 10.1186/s11671-017-2236-0

**Published:** 2017-08-01

**Authors:** Mahbubur Rahman, Lee Yook Heng, Dedi Futra, Tan Ling Ling

**Affiliations:** 1 Department of General Educational Development (GED), Faculty of Science and Information Technology, Daffodil International University, 102 & 102/1, Shukrabad, Mirpur Road, Dhanmondi, Dhaka, 1207 Bangladesh; 2 School of Chemical Sciences and Food Technology, Faculty of Science and Technology, University Kebangsaan Malaysia, Bangi, 43600 UKM Selangor D.E. Malaysia; 3 Southeast Asia Disaster Prevention Research Initiative (SEADPRI-UKM), Institute For Environment and Development (LESTARI), University Kebangsaan Malaysia, Bangi, 43600 UKM Selangor D.E. Malaysia; 4 Department of Chemistry Education, Faculty of Education, Graduate Program, University Riau, Pekanbaru, Riau 28131 Indonesia

**Keywords:** *Vibrio cholerae*, Polystyrene co-acrylic acid (PSA) particles, Latex-gold nanosphere, Biosensors, Electrochemical determination, EDC/NHS chemistry

## Abstract

An ultrasensitive electrochemical biosensor for the determination of pathogenic *Vibrio cholerae* (*V*. *cholerae*) DNA was developed based on polystyrene-co-acrylic acid (PSA) latex nanospheres-gold nanoparticles composite (PSA-AuNPs) DNA carrier matrix. Differential pulse voltammetry (DPV) using an electroactive anthraquninone oligonucleotide label was used for measuring the biosensor response. Loading of gold nanoparticles (AuNPs) on the DNA-latex particle electrode has significantly amplified the faradaic current of DNA hybridisation. Together with the use of a reported probe, the biosensor has demonstrated high sensitivity. The DNA biosensor yielded a reproducible and wide linear response range to target DNA from 1.0 × 10^−21^ to 1.0 × 10^−8^ M (relative standard deviation, RSD = 4.5%, *n* = 5) with a limit of detection (LOD) of 1.0 × 10^−21^ M (*R* ^2^ = 0.99). The biosensor obtained satisfactory recovery values between 91 and 109% (*n* = 3) for the detection of *V*. *cholerae* DNA in spiked samples and could be reused for six consecutive DNA assays with a repeatability RSD value of 5% (*n* = 5). The electrochemical biosensor response was stable and maintainable at 95% of its original response up to 58 days of storage period.

## Background

*Vibrio cholerae*, a food-borne pathogen, can cause epidemics of cholera in human through the condition of acute watery diarrhoea. Cholera outbreak is still a serious problem in some parts of the world, e.g. Asia and Africa, and leads to low socio-economic status [1–4]. This enteric pathogen is a leading cause of morbidity and mortality, particularly in developing countries [5]. The epidemic and pandemic of cholera in various regions are mainly caused by *V. cholerae* serogroups O1 and O139 [1, 2, 6]. *V. cholerae* serogroup O1 has two major serotypes, i.e. Inaba and Ogawa, which may alternate among cholera epidemics. The third serotype, Hikojima, also exists but is rare and unstable. The genes responsible for O1 antigen biosynthesis have been designated as rfb. The mutation which defines serotypes Inaba and Ogawa is a single deletion mutation in the rfbT gene [7]. However, occasional food-borne outbreaks in humans with severe diarrhoea have also been reported to be caused by non-O1/non-O139 *V. cholerae* through the ingestion of undercooked seafood [8] or exposure to a contaminated aquatic environment [9]. The first epidemic of *V. cholerae* O139 occurred in 1992 in Bangladesh and India and then it spread rapidly to other countries in Southeast Asia [10]. Worldwide, in 2005, a total of 131,943 cholera cases and 2272 deaths were reported to the World Health Organization (WHO) [11].

The quest for an effective method for monitoring or diagnosis of toxigenic *V. cholerae* bacteria is imperative to control the cholera epidemic. Traditional identification of *V. cholerae* is often achieved through the isolation and screening of the bacteria, where it involves pre-enrichment in alkaline peptone water (APW) followed by isolation of *V. cholerae* on the thiosulfate citrate bile salt sucrose agar (TCBS) culture medium and identification by slide agglutination test with specific antisera [12]. However, this technique is very time-consuming and labour-intensive, and the result obtained several days later would have meant a delay in clinical diagnosis and patient treatment. Molecular method involving PCR amplification for the detection of *V. cholerae* [13] has reduced the diagnosis time [14], but PCR method requires skilled professional and expensive infrastructure that is difficult to perform in countries with low resource setting. Rapid diagnostic tests based on immunochromatography principle have been reported for discrete or simultaneous detection of *V. cholerae* serogroups O1 and O139. Some other immunoassay-based techniques used for the detection of *V. cholerae* are such as enzyme-linked immunosorbent assay (ELISA), coagglutination, immunofluorescence, and quartz crystal microbalance (QCM). However, most of these techniques require sophisticated instrumentation, long assay time, and highly qualified personnel with detailed technical knowledge [15–20].

Electrochemical methods have attracted considerable attention in nucleic acid detection due to their high sensitivity, specificity, simplicity, and economical protocol, as well as rapid detection and compatible with microfabrication technology [21, 22]. Additionally, electrochemical method that is coupled with miniaturisation technologies can be used for in situ decentralised analysis, for instance, the microfluidic chip-based DNA biosensor device, which is very useful for practical setting [23]. There is a wide range of electrodes used in the electrochemical measurements such as glassy carbon electrode (GCE), carbon paste electrode (CPE), gold electrode, and platinum electrode. Recently, studies have been concentrated towards the use of screen-printed electrodes (SPEs) due to some of their unique properties such as providing low background current and broad potential window, cost effective as the carbon ink is inexpensive, and can be mass-produced.

There are a few electrochemical methods reported for the detection of *V. cholerae* consisted of a series of complex steps. An electrochemical *V. cholerae* genosensor reported by Liew et al. [24] used electrochemical adsorption method to immobilise the DNA probe on the carbon SPE. The lyophilised AuNPs-modified multilayer PSA particles with polyelectrolytes formed bioconjugate with avidin to function as the reporter label in the sandwich DNA hybridisation assay. However, the addition of sorbitol stabiliser was needed to preserve the PSA-AuNPs-avidin bioconjugates to lengthen the operational period of the DNA biosensor up to 30 days. Enzymatic electrochemical *V. cholerae* DNA biosensor has been recently devised by Yu et al. [25], whereby the thiolated anti-fluorescein-conjugated alkaline phosphatase (anti-FCAP)-labelled DNA probe was bound to the gold SPE through gold-thiol chemistry. The target DNA was tagged with a universal fluorescein to allow DNA hybridisation recognition achieved via enzymatic conversion of α-naphthyl phosphate to electroactive α-naphthol. Nevertheless, this detection scheme required a long assay time of approximately 95 min for DNA hybridisation, labelling of the DNA hybrids with functional enzyme followed by incubation of the electrode in electroinactive α-naphthyl phosphate substrate before an amperometric measurement can be made. Another enzymatic electrochemical DNA biosensor based on avidin-coated carbon SPE conjugated with biotinylated DNA probe was developed by Low and team members [26]. A digoxigenin (DIG)-labelled reporter probe was also used in this double hybridisation strategy that flanked the cDNA sequence. Horseradish peroxidase-linked anti-DIG (anti-DIG-HRP) was employed as the electrochemical label, which could concurrently catalyse the oxidation of 3,3′,5,5′-tetramethylbenzidine (TMB) with the reduction of H_2_O_2_ to yield an electron transfer to the electrode surface for electrochemical transduction of DNA hybridisation event. A facile DNA biosensor design based on thiolated DNA probe immobilised gold-coated glass electrode was described by Patel et al. [22] for rapid detection of *V. cholerae*, and methylene blue was used as the DNA hybridisation indicator. However, the linear detection range of the system is confined at μM levels, which limited its application in clinical samples.

Latex-gold nanoparticle has been previously employed as the DNA hybridisation label via avidin/biotin binding to the DNA probe in the detection of *V. cholerae* [24], fish pathogen *Aphanomyces invadans* [27], *E. coli* [28], and nonspecific DNA hybridisation [29], whereby the latex spheres were coated with a multilayer of polyelectrolyte before the negatively charged colloids of gold nanoparticles were electrostatically attached thereunto. Kawde and Wang [29] attached the PSA latex particles to a DNA reporter probe to be used as DNA hybridisation label via loading of streptavidin-coated latex particles with biotin-coated AuNPs. Kuan et al. [24, 27] and Liew et al. [24, 27] also reported the same avidin-biotin binding method using gold-PSA-DNA probe conjugates. Pinijsuwan et al. [28] reported the use of electrostatic method for the loading of PSA particles attached to DNA reporter probe and the polyelectrolyte gold-coated PSA particles were used as a label for hybridisation that amplified the DPV current response.

In this study, we are reporting a different DNA immobilisation approach using the latex-gold nanoparticles as the DNA probe immobilisation substrate to develop a highly sensitive detection system for *V. cholerae* DNA. Immobilisation of DNA was performed with a very simple and fast procedure using 1-ethyl-3-(3-dimethylaminopropyl) carbodiimide hydrochloride/N-hydroxysuccinimide (EDC/NHS) chemistry as coupling reagent for improving immobilisation efficiency [30] on the carboxylated latex particle surface. The DNA hybridisation detection was based on the sandwich-type assay, which involved hybridisation reaction between immobilised DNA probe and target sequence followed by a signal/reporter probe. Anthraquinone-2-sulfonic acid mono-hydrate sodium salt (AQMS) was employed as an electrochemical label to monitor the hybridisation event. The proposed sub-micron-sized latex particles improved the DNA probe binding capacity, and the sensitivity of the DNA biosensor was enhanced with the incorporation of the highly conductive gold nanoparticles (AuNPs). The DNA biosensor demonstrated exceptional sensitivity to the detection of *V. cholerae* cDNA and extremely low detection limit at zeptomolar levels compared to avidin-biotin technology reported so far [24, 26].

## Methods

### Chemicals and Reagents

Styrene (St) and acrylic acid (AA) were purchased from Fluka. HAuCl_4_·3H_2_O, trisodium citrate dehydrate, sodium dodecyl sulphate (SDS), 1-ethyl-3-(3-dimethylaminopropyl) carbodiimide hydrochloride (EDC), and N-hydroxysuccinimide (NHS) were obtained from Sigma-Aldrich. Ammonium persulfate (APS), hydrobromic acid, and bromine were supplied by Riedel-De Haën, Ajax Finechem, and Panreac, respectively. All the chemical solutions were prepared with deionised water. Both 30-base target and mismatch synthetic oligonucleotides were procured from Bio Service Unit (NSTDA). Non-complementary DNA (ncDNA) and signal probe were from Sigma and the 5′-amino-modified capture probe was from Bioneer. The capture probe was prepared in 0.05 M of potassium phosphate buffer (pH 7), while target DNA, mismatched target, reporter probe, and non-complementary DNA solutions were prepared in sodium phosphate buffer (0.05 M, pH 7). The oligonucleotide sequences used in the present study are shown in Table 1.

**Table 1:** The list of DNA sequences used in this study

Oligonucleotide	Base sequence
DNA capture probe	5′-TCA TCG ACC TGT AAG-3′ [AmC3]
Linear target sequence	5′-TCA AAC CGT GCT GAA CTT ACA GGT CGA TGA-3′
3 bases mismatched DNA	5′-TCA AAC CGT GCT GAA CTT GTC GGT CGA TGA-3′
Non-complementary strand	5′-CGT GGT TTT ACC ATT TGC AAC AGC A-3′
Reporter probe	5′-TTC AGC ACG GTT TGA

### Apparatus

The electroanalytical measurement was performed with a potentiostat/galvanostat (Autolab PGSTAT12, Metrohm) equipped with GPES (4.0.007) software. The voltammetric experiments were carried out with a conventional three-electrode system consisting of a carbon paste screen-printed working electrode (SPE) (Quasense, Bangkok, Thailand), Ag/AgCl reference electrode (3 M of KCl), and platinum rod (2 mm diameter) counter electrode. Differential pulse voltammetry (DPV) technique was used for all electro-chemical investigations at 0.02 V step potential and 0.5 V/s scan rate from 0.0 to +1.0 V in 4.5 mL measuring buffer (0.05 M of potassium phosphate buffer) at pH 7 and ambient temperature. All the potentials measured in this study were referred to Ag/AgCl electrode, and homogeneous solutions were prepared using a sonicator bath (Elma S30H). A scanning electron microscope (SEM, LEO 1450VP) was used to determine the size and distribution of the latex spheres.

### Methods

#### Synthesis of Colloidal Gold Nanoparticles

Colloidal AuNPs were prepared by sodium citrate reduction following Turkevich method [31]. Briefly, about 10 mL of 5 mM of HAuCl_4_·3H_2_O was dissolved in 180 mL of deionised water and boiled under continuous stirring condition on a combined hot plate-magnetic stirrer device. Ten millilitres of 0.5% (*w*/*v*) of trisodium citrate was then added into the boiling solution, and the colour of the solution was observed to gradually change from pale red to ruby red.

#### Preparation of Latex Particles

Latex particles were prepared by soap-free emulsion copolymerisation reaction as described by Polpanich et al. [23] with some modifications. In brief, about 190 g of deionised water was purged with nitrogen gas in a three-neck flask submerged in a water bath for ∼1 h under stirring at 350 rpm. Twenty grams of St and 0.5 g of AA were then added, and the temperature was maintained at 70 °C. After that, about 0.2 g of APS was added into 10 mL of deionised water followed by pouring into the formulation in the three-neck flask to initiate the radical polymerisation reaction, and the polymerisation process was proceeded for 8 h. The as-synthesised carboxyl latex spheres were harvested by centrifugation with deionised water twice at 13,000 rpm for 20 min [23, 27, 28] and re-suspended in deionised water at room temperature (25 °C) until use. The morphology and average size of the PSA latex particles were determined by scanning electron microscopy (SEM).

#### Modification of SPE Surface and DNA Probe Immobilisation

**Fig. 1:**
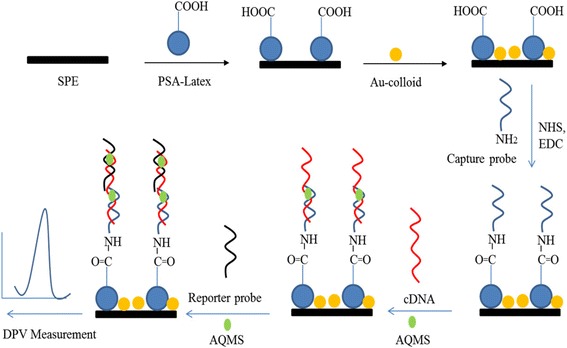
Schematic representation of DNA biosensor stepwise fabrication procedure

Prior to surface modification, the carbon SPE was rinsed thoroughly with deionised water and then drop-coated with the PSA suspension at 3 mg/mL and allowed to air-dry at ambient conditions followed by drop-casting with 5 mg/mL of colloidal AuNPs. The electrochemical characteristics of carbon SPE before and after modification with latex particles and AuNPs were examined with CV method. The latex-AuNPs-modified carbon SPE (PSA-AuNPs-SPE) was then rinsed with deionised water and immersed in 0.1 M of potassium phosphate buffer (pH 5) containing carbodiimide cross-linking reagents, i.e. 0.002 M of EDC and 0.005 M of NHS for 2 h [32] before soaking overnight in 0.05 M of potassium phosphate buffer (pH 7) containing 5 μM of capture probe. After that, the DNA-modified PSA-AuNPs-SPE (DNA-PSA-AuNPs-SPE) was thoroughly washed with potassium phosphate buffer (0.05 M, pH 7) to remove the physically adsorbed probe. The DNA electrode was immersed in 0.05 M of sodium phosphate buffer at pH 7 containing linear target DNA (1 μM) and AQMS DNA hybridisation label (5 mM) for partial hybridisation for 1 h and later dipped in 0.05 M of sodium phosphate buffer (pH 7) conditioned with 1 μM of reporter probe and 5 mM of AQMS for another 1 h for full DNA hybridisation process. Finally, the DNA electrode was rinsed with potassium phosphate buffer (0.05 M, pH 7) for the removal of non-hybridised oligonucleotide sequences. The electrochemical response of each extended substance attached on the SPE was investigated with DPV method. Figure 1 represents the stepwise procedure for the development of *V. cholerae* DNA biosensor based on colloidal PSA-AuNPs solid support.

#### Optimization of Synthetic Oligonucleotide Hybridization

The effect of various parameters such as DNA probe and AQMS concentrations, pH, ionic strength and buffer capacity on the hybridisation response of the immobilised DNA probe with signal probe and target DNA has been examined prior to the determination of the dynamic linear range of the DNA biosensor. Moreover, capture probe immobilisation and DNA hybridisation durations as well as biosensor lifetime and regeneration were also assessed before the developed *V. cholerae* DNA biosensor was ready for the application in spike-and-recovery experiment. Capture probe and AQMS loadings were optimised by varying their concentrations from 1 to 6 μM and 0.1–5 mM, respectively, while the concentration of target DNA and reporter probe was maintained at 5 μM in 0.05 M of sodium phosphate buffer (pH 7.0). pH effect and buffer concentration studies were conducted by changing the pH and concentration of sodium phosphate buffer from pH 5.5 to 8.0 and 0.001 to 1.000 M, respectively. The presence of different cations in the DNA hybridisation response of the electrochemical DNA biosensor was performed by adding Na^+^, K^+^, Ca^2+^, and Fe^3+^ ions at 1.0 M into the DNA hybridisation buffer containing 1 mM of AQMS and 5 μM of cDNA and detection probe of pH 7.0. Ionic strength effect was examined by varying the NaCl concentration over the range of 0.1–3.0 M at pH 7.0. Dynamic range of the DNA biosensor was then determined in 1.0 × 10^−21^ to 1.0 × 10^−8^ M *V. cholerae* cDNA using a constant signal probe concentration at 5 μM and pH 7.0. DNA probe immobilisation duration was determined by soaking the DNA electrode into 5 μM of capture probe solution (pH 7.0) between 1 and 13 h, and the DPV response was taken every 1–2 h. Meanwhile, the DNA hybridisation time was determined by permitting the DNA hybridisation reaction to occur between 15 and 180 min, and the DNA biosensor response was recorded every 15–30 min. The shelf life of the DNA biosensor was determined by periodically measuring the DNA biosensor response towards the detection of 5 μM *V. cholerae* cDNA for 120 days. The analysis was conducted in five replicates using new DNA electrode for each sandwich hybridisation assay. Regeneration of the DNA electrode was done using 0.1 M of NaOH solution for 4 min, and rehybridisation of the DNA biosensor (60 min) was accomplished using rehybridisation solution containing 5 μM of cDNA and detection probe and 1 mM of AQMS at 2.0 M ionic strength in 0.05 M of potassium phosphate buffer (pH 7.0). The regeneration experiment was conducted in six replicates.

#### *V. cholerae* Quantification Using PSA-AuNPs-Based Electrochemical DNA Biosensor

Various *V. cholerae* bacterial strains namely J2126-I, J2126-II, J3324-I, J3324-II, J3330-I, J3330-II, CDHI 5294-II, and UVC1324 including *Citrobacter freundii* (CF-I) and *Citrobacter freundii* (CF-II) were obtained from Microbiology Laboratory, Faculty of Applied Sciences, AIMST University, Kedah. Genomic DNA extraction was then conducted over these bacteria using QIAGEN Genomic-tip 500/G according to the manufacturer’s protocol. The extracted DNA was then diluted 100-fold using sodium phosphate buffer (0.05 M, pH 7.0). About 300 mL of the extracted DNA containing 2.0 M of NaCl and 1 mM of AQMS was sonicated for 15 min to release the DNA breaks. Then, the immobilised DNA probe was soaked for 1 h to allow DNA hybridisation process to take place and washed carefully with 0.05 M potassium phosphate buffer (pH 7.0) to remove the unbound DNA. Evaluation of the DNA biosensor response based on DPV peak current was measured and compared with the current response generated by the DNA electrode without reaction with cDNA as a control signal. Each experiment was carried out in triplicate under the same experimental conditions. Common *t* test was used to determine significant difference between the DNA biosensor response and the control’s response. The recovery of *V. cholerae* J3324 and *V. cholerae* UVC1324 DNAs at 1.0 × 10^−4^ μg μL^−1^, 1.0 × 10^−5^ μg μL^−1^, and 1.0 × 10^−6^ μg μL^−1^ spiked into the hybridisation buffer was then carried out using the proposed PSA-AuNPs-based electrochemical DNA biosensor.

## Results and Discussion

### Morphology of Latex Particles and Electrochemical Behaviour of Latex-Gold Nanoparticle Modified SPE.

**Fig. 2:**
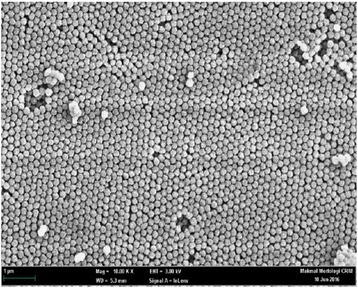
SEM micrograph of the as-synthesised PSA latex spheres at 10,000 magnifications

Figure 2 shows the SEM micrograph of the carboxylated latex spheres with an average particle size of 186.1 ± 4.6 nm. The uniform size distribution of the PSA spheres allowed homogenous immobilisation of DNA molecules on the latex surface to enhance the reproducibility response of the DNA biosensor. A scanning electron microscope (SEM, LEO 1450VP) was used to determine the size and distribution of the latex spheres.

The electrodynamic results of the modified SPE are tabulated in Table 2. The peak potential separation (ΔEp) indicates the electron transfer kinetics of the system. The PSA-modified SPE (PSA-SPE) showed the highest ΔEp value due to the slow charge transfer process in the colloidal copolymer particle layer, which rendered the system moving towards quasi-reversible state. However, when the AuNPs were loaded on the PSA-SPE, the decrement in the ΔEp implies an improvement on the electron transfer rate at the electrode surface.

**Table 2:** Electrochemical data acquired from the CVs of carbon SPE before and after modification with PSA latex particles and AuNPs

Electrode	Epa (V)	Epc (V)	ΔEp (V)	Ipa	Ipc	Ipa/Ipc
Bare SPE	0.545	−0.243	0.788	5.06E-06	−6.73E-06	−0.75
AuNPs-SPE	0.407	−0.213	0.620	1.45E-05	−2.765E-05	−0.52
PSA-SPE	0.573	−0.286	0.859	5.96E-06	−6.83E-06	−0.87
PSA-AuNPs-SPE	0.52	−0.276	0.796	3.04E-05	−4.20E-05	−0.72

**Fig. 3:**
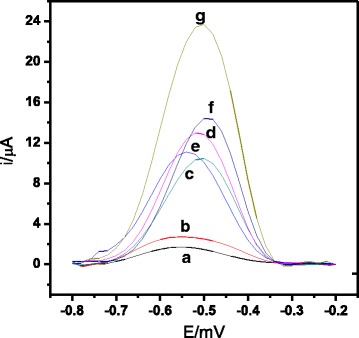
Differential pulse voltammograms signal from AQMS of the electrodes (**a**) PSA-SPE, (**b**) PSA-AuNPs-SPE, (**c**) capture probe-PSA-AuNPs-SPE and when in the presence of (**d**) ncDNA and reporter probe, (**e**) mismatch DNA and reporter probe, (**f**) cDNA alone, and (**g**) cDNA and reporter probe

Figure 3 shows the differential pulse voltammograms of AQMS oxidation response on the latex-modified SPE and the sequential hybridisation response of the *V. cholerae* DNA biosensor. Significant DPV peak current difference was observed between electrodes that contained latex-modified microsphere only and without immobilised capture DNA (experiments (a) and (b)) and modified with immobilised capture DNA probes in the presence of cDNA and reported probe (experiment (g)). This indicates that the aminated DNA capture probes were successfully immobilised onto the coated carboxylated PSA latex spheres via EDC/NHS coupling protocol [33]. Experiment (g) also shows a much higher DPV response compared with DNA electrodes in the presence of ncDNA and reported reporter probe (experiment (d)), in mismatched DNA and reported reporter probe (experiment (e)),, and target cDNA with no reported probe (experiment (f)). This is due to the complete hybridisation of the target DNA with capture and reporter probes through sandwich hybridisation reaction on the DNA biosensor surface as demonstrated in experiment (g). This also shows that the use of reported probe could enhance the signal from DNA hybridisation. Nevertheless, the DPV current resulted from hybridization observed in the presence of target DNA without incorporation of a reported probe (experiment (f)) is still higher than the DPV current signals observed for unhybridised DNA (experiments (c), (d), and (e)).

### Effect of DNA Probe Loading and AQMS Concentration

**Fig. 4:**
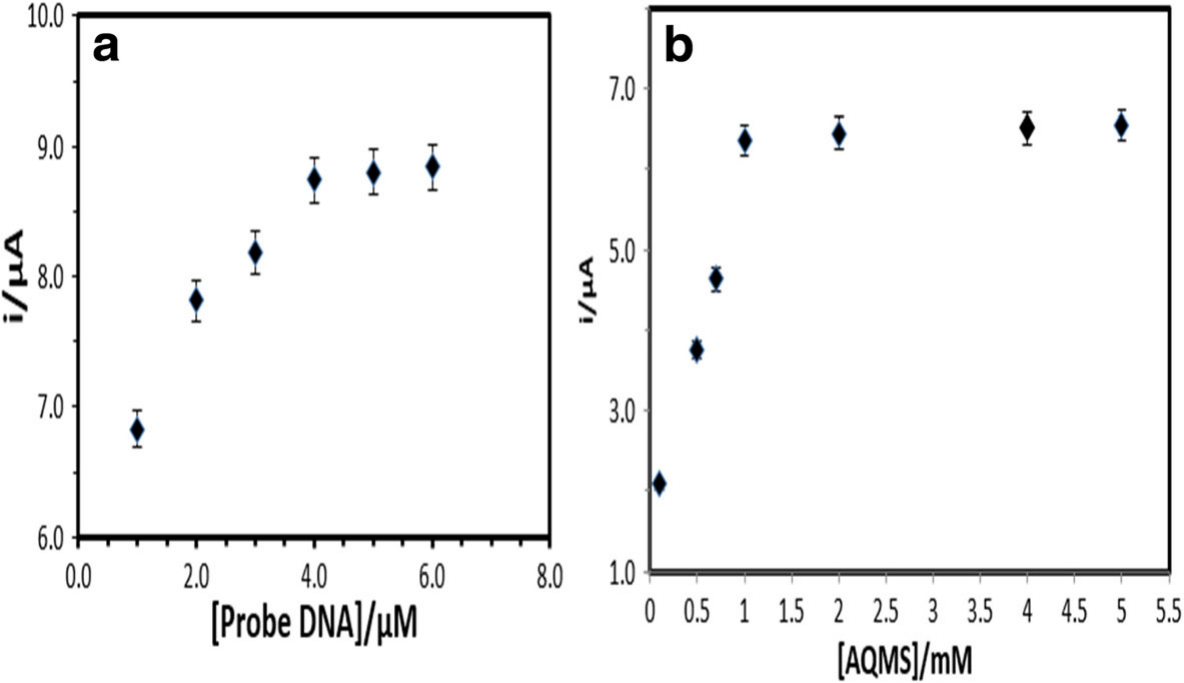
The effect of capture probe (**a**) and AQMS concentrations (**b**) on the DNA biosensor response performed with 5 μM cDNA and signal probe in 0.05 M sodium phosphate buffer (pH 7.0)

The effect of DNA probe concentration on the DNA hybridisation response was observed through the AQMS electrochemical oxidation response. Figure 4a shows that the DNA biosensor response increased gradually with the increasing of the DNA probe amount immobilised on the PSA-AuNPs-SPE from 1 to 4 μM. This was attributed to the increasing amount of electroactive AQMS intercalated in the double-stranded DNA (dsDNA) to render an electron transfer through the immobilised DNA helix. The DPV response of the DNA biosensor was observed to become almost plateau between 4 and 6 μM DNA probe, which indicates an optimum DNA probe loading on the electrode surface was achieved [34]. Therefore, 4 μM capture probe was selected as an optimum DNA probe loading in the subsequent experiments. The concentration of AQMS label has also been optimised in the measuring electrolyte between 0.1 and 5.0 mM, and the concentration of AQMS at 1 mM was found to be sufficient for optimum DNA intercalation reaction (Fig. 4b).

### Effect of pH, Ionic Strength, and Buffer Capacity

**Fig. 5:**
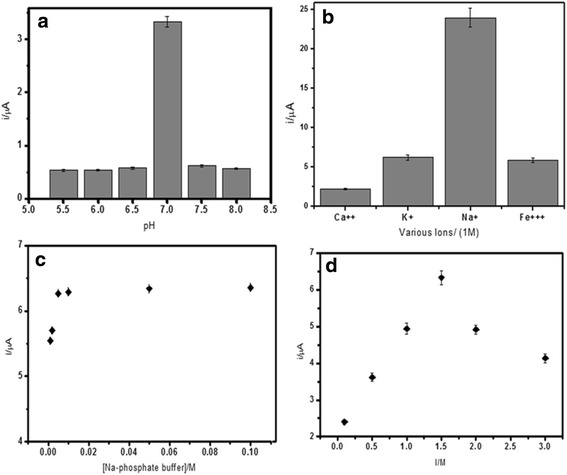
The effect of pH (**a**) various cations (**b**), buffer concentration (**c**), and ionic strength (**d**) on the DNA hybridization response of the electrochemical *V. cholerae* DNA biosensor. Hybridization was performed with 5 μM cDNA and reporter probe followed by intercalation with 1 mM AQMS

The rate of DNA hybridisation reaction is very much dependent on the solution pH. As can be seen in Fig. 5a, under a more acidic environment, the protonation of phosphodieter backbone of DNA reduced the solubility of the DNA molecule, which eventually decreased the DNA hybridisation reaction rate. Whereas in basic medium, it broke the weak hydrogen bonds holding DNA base pairs together. Optimum DNA hybridisation reaction was more favourable in neutral condition, whereby it promoted more capture probes to hybridise with target DNA and subsequently allowed intercalation of AQMS redox probes to make the DNA hybridisation recognition into business. Thus, 0.05 M of sodium phosphate buffer at pH 7.0 was used as the DNA hybridisation medium for subsequent DNA biosensor studies. Positively charged ions such as Ca^2+^, Na^+^, K^+^, and Fe^3+^ ions can interact with the negatively charged phosphodiester chain of DNA. This ionic reaction will neutralise the charge of DNA molecule, thus decreasing the steric repulsions between DNA molecules to ease the DNA hybridisation reaction. Figure 5b depicts the effect of some cations on the DNA hybridisation response. The DNA hybridisation response was noticed to increase in the presence of positively charged ion in the order of Na^+^ > K^+^ > Fe^3+^ > Ca^2+^. Both Ca^2+^ and Fe^3+^ ions were found to considerably reduce the DNA hybridisation response because of the ionic interactions of Ca^2+^ and Fe^3+^ ions with phosphate ions from the buffer solution, which led to the formation of insoluble phosphate compounds. This has reduced the ionic content of the medium, thereby increasing the electrostatic repulsion between DNA molecules. The highest DNA hybridisation current was obtained in the presence of Na^+^ ion due to its smaller size and stronger affinity towards DNA sugar-phosphate backbone compared to K^+^ ion to overcome the steric hindrance and electrostatic repulsion between negatively charged phosphate groups of DNAs.

Additionally, ionic strength of the solution would also affect the DNA biosensor response. Figure 5c, d shows the optimum buffer capacity, and ionic strength were achieved using 0.05 M of sodium phosphate buffer with the pH fixed at pH 7.0 and 2.0 M of NaCl, respectively. In this condition, it favoured the DNA hybridisation reaction to the highest degree; hence, high DPV response was yielded. At optimum buffer capacity and ionic strength of the solution, the electrostatic repulsion between DNA molecules decreased and thus improving the DNA hybridisation reaction. In contrast, when too low or too high ionic content was used, steric hindrance and electrostatic repulsion became dominant and restricted the hybridisation of DNA molecules.

### Establishment of *V. cholerae* DNA Biosensor Calibration Curve

**Fig. 6:**
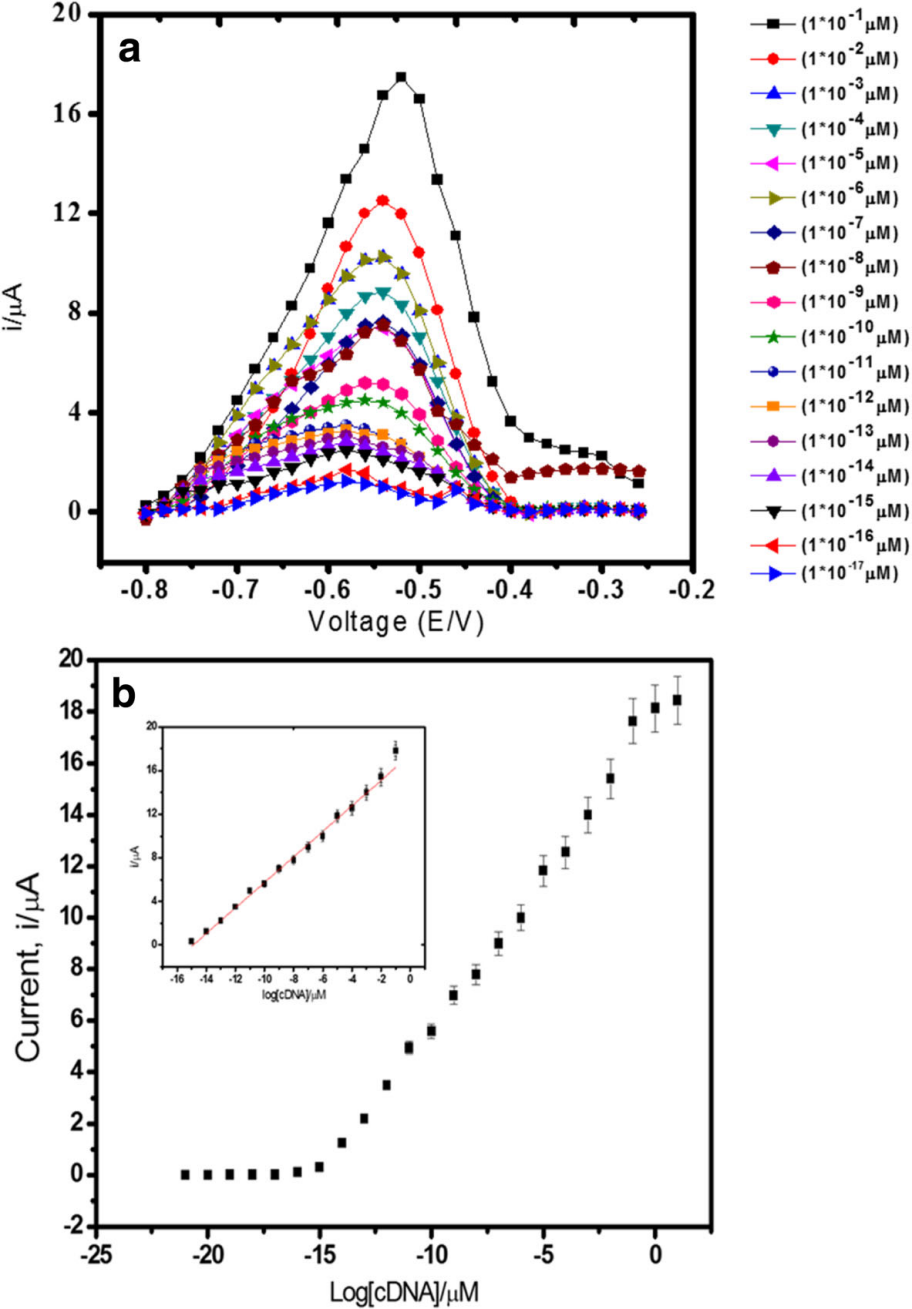
Differential pulse voltammograms (**a**) and DNA biosensor linear range (**b**) obtained using various cDNA concentrations from 1.0 × 10^−15^ to 1.0 × 10^−1^ μM *V. cholerae* target DNA and 5 μM signal probe

From the result shown in Fig. 6a, the DNA biosensor response increased proportionally with the increasing cDNA concentration from 1.0 × 10^−21^ to 1.0 × 10^−8^ M (*R* ^2^ = 0.99) with a limit of detection of 1.0 × 10^−21^ M. The detection limit was calculated based on three times the standard deviation of the biosensor response at the response curve approximating the limit of detection divided by the linear calibration slope. The broad linear detection range of the DNA biosensor was due to the highly monodispersed and spherical PSA latex particles in submicron-sized range used as the carrier matrix for DNA immobilisation. The acrylic acid-rich layer on the latex particles surface offered a large binding site for attachment of DNA capture probes to create a maximal covered surface by the DNA receptive layer. In addition, the incorporation of AuNPs on the PSA-modified SPE further amplified the analytical signal of the DNA hybridisation response, and this rendered high sensitivity of the DNA biosensor (Fig. 6b).

### DNA Probe Immobilisation and Hybridization Times

**Fig. 7:**
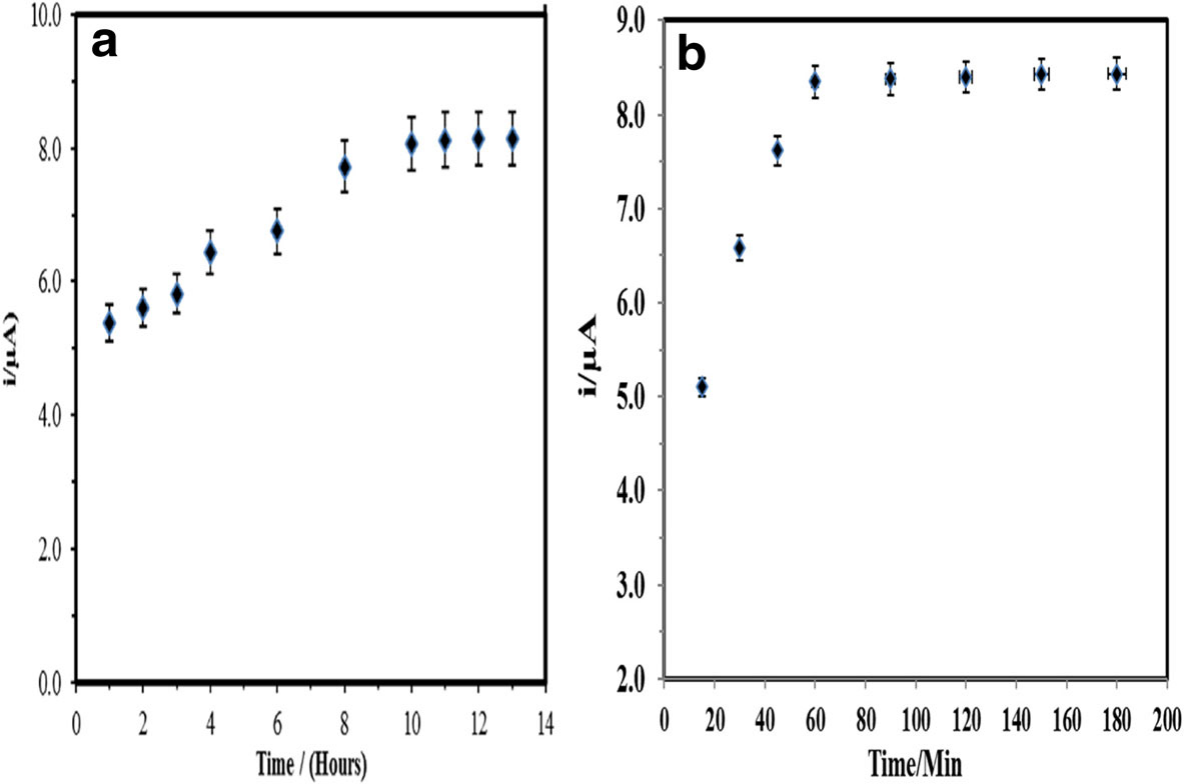
DNA probe immobilisation duration on the immobilised PSA latex colloidal particles (**a**) and DNA hybridization duration of the DNA biosensor (**b**) in 0.05 M potassium phosphate buffer at pH 7.0 containing 5 μM target DNA and reporter probe and 1 mM AQMS at 2.0 M ionic strength

It took about 8 h for the capture probe to be immobilised on the PSA copolymer particles surface, as illustrated by the DNA biosensor response in Fig. 7a, which showed a current increment from 1.0 to 8.0 h of capture probe immobilisation time, after which no obvious change in the DPV current was observed. Longer immobilisation time resulted in a higher amount of DNA probes immobilised onto the latex. After 8.0 h of exposure to the DNA probes, the hydrophilic functional latex with reactive carboxyl groups at the surface was presumably fully attached with the DNA probes. DNA hybridisation time, on the other hand, is the rate limiting step, which determines the response time of the DNA biosensor. Based on the DNA biosensor response trend in Fig. 7b, the response time of the *V. cholerae* DNA biosensor developed in this study was estimated to be about 60 min for the dual hybridisation processes to complete.

### Long-Term Stability and Regeneration of *V.cholerae* DNA Biosensor

**Fig. 8:**
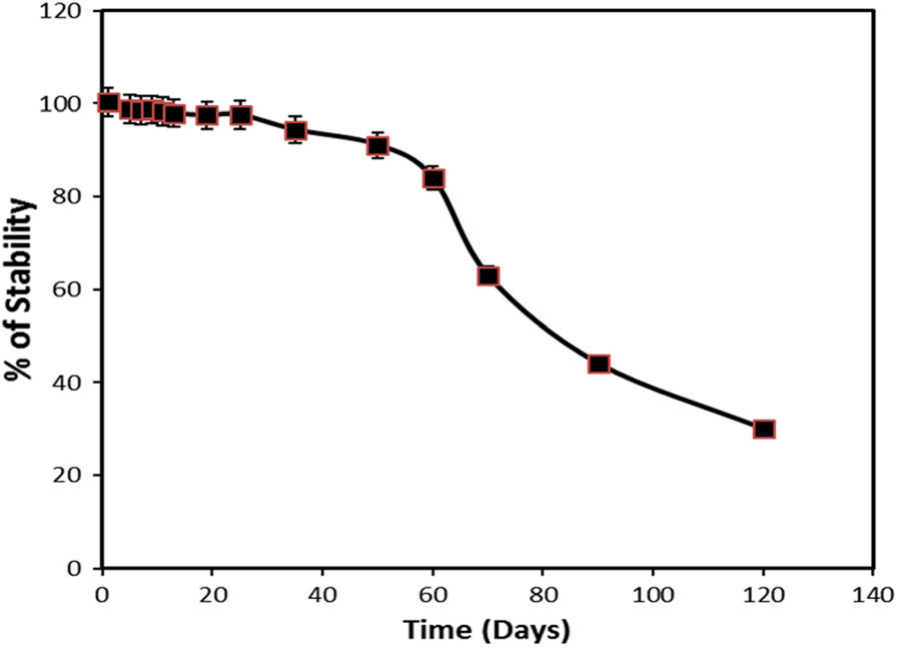
The life span profile of the fabricated *V. cholerae* DNA sensing electrode. The electrode was stored in 0.05 M potassium phosphate buffer (pH 7.0) at 4 °C after every DPV measurement was taken

Figure 8 shows the shelf life of the *V. cholerae* DNA biosensor. The DNA biosensor showed the highest response to the detection of 5 μM of *V. cholerae* cDNA for the first month of the experimental period. The electrochemical DNA biosensor was able to retain 95% of its initial DPV current after 58 days of storage period. The DNA hybridisation response was then gradually decreased to about 75% of its original response on the 75th day and exhibited 40% of its initial performance on the 100th operational day. The bioactivity of the immobilised capture probe was finally declined to 30% after 3 months of storage period. The reproducibility of each calibration point, which was repeated on five replicate DNA electrodes, gave satisfactory relative standard deviation (RSD) between 2.4 and 4.5% (*n* = 5).

**Fig. 9:**
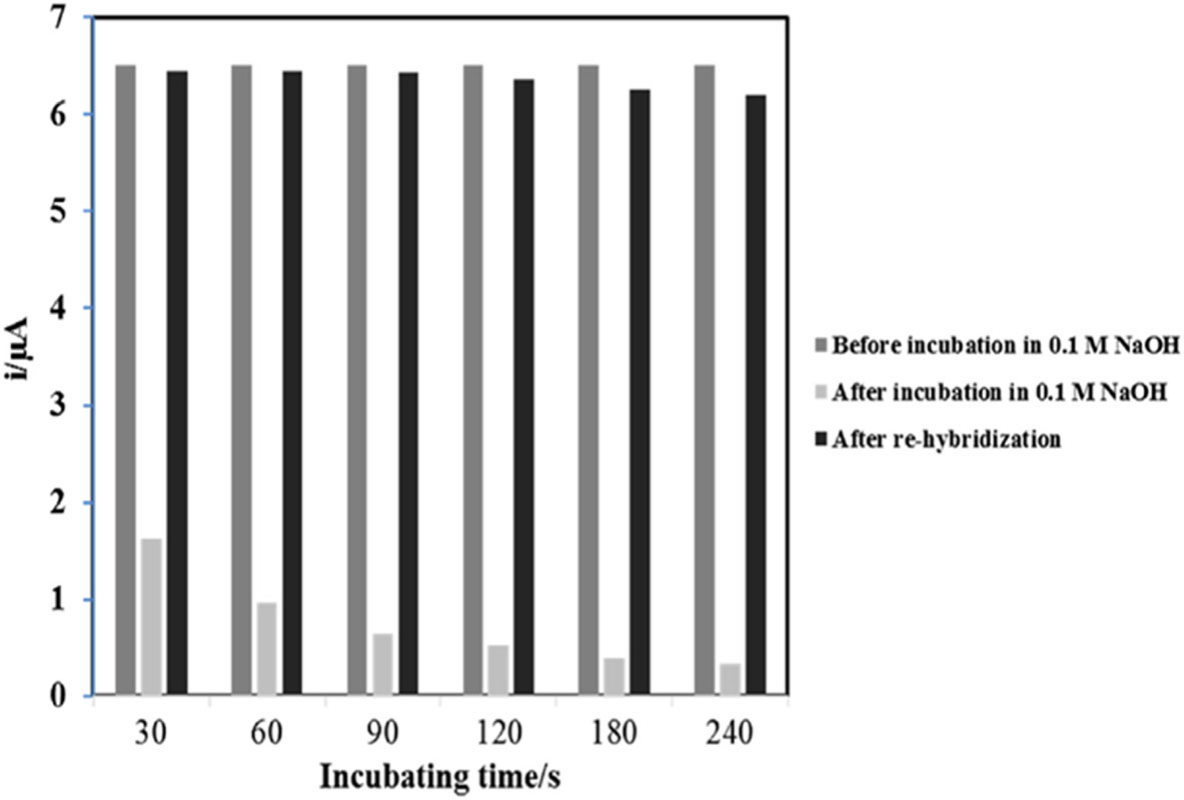
Repeatability of *V. cholerae* DNA biosensor using 0.1 M NaOH regeneration solution and rehybridization solution containing 5 μM cDNA and detection probe and 1 mM AQMS at 2.0 M ionic strength in 0.05 M potassium phosphate buffer (pH 7.0)

Regeneration of biosensor indicates whether the biosensor is reusable for a series of consecutive analyses. The regeneration method used in this study was conducted based on previously reported protocol in other studies [11, 21] with slight modifications. In this study, 0.1 M of NaOH solution was used as the regeneration solution to break the hydrogen bonds between base pairs of hybridised dsDNA. With the result from Fig. 9, it is notable that the DNA biosensor response declined significantly after incubation in 0.1 M of NaOH and the percentage of the DNA biosensor response reduced from 35.1 to 5.2% relative to the DNA biosensor initial response after incubation in 0.1 M of NaOH solution from 30 to 240 s. The DNA biosensor response decreased with the increasing incubation time signifies the hydrogen bonds between hybridised dsDNA were broken up by the alkaline regeneration solution. However, rehybridisation of the DNA biosensor was able to attain almost 100% of its initial response for a consecutive six DNA analyses with a reversibility RSD of 5%.

### Determination of *V. cholerae* Bacteria with the Developed DNA Biosensor

The optimised DNA biosensor has been applied to quantify the *V. cholerae* DNA extracted from various *V. cholerae* bacterial strains. Table 3 presents the results acquired from DNA tests carried out with the hybridisation medium spiked with different strains of *V. cholerae* DNAs and other bacterial species at a concentration within the calibration range of the DNA biosensor. The DNA biosensor showed superior selectivity towards *V. cholerae* J3324–I, *V. cholerae* J3324–II, and *V. cholerae* UVC1324 with high DPV current response and low current signals were obtained for the evaluation of both *Citrobacter freundii* (CF-I) and *Citrobacter freundii* (CF-II).

**Table 3:** Selectivity of DNA biosensor towards the detection of *V. cholerae* DNA

Real samples	(DNA) (μg/μL)	Current ± SD (μA)	Control ± SD (μA)	*t* test	Current (%)	Remark
*Citrobacter freundii* (CF-I)	1.0 × 10^−4^	0.250 ± 0.01	0.261 ± 0.02	1.115	2.27	×
*Citrobacter freundii* (CF–II)	1.0 × 10^−4^	0.248 ± 0.06	0.261 ± 0.02	0.267	2.26	×
*Vibrio cholerae* J2126-I	1.0 × 10^−4^	2.568 ± 0.09	0.261 ± 0.02	7.561	23.37	√
*Vibrio cholerae* J2126-II	1.0 × 10^−4^	2.579 ± 0.10	0.261 ± 0.02	7.983	23.47	√
*Vibrio cholerae* J3324-I	1.0 × 10^−4^	10.117 ± 0.20	0.261 ± 0.02	15.630	92.06	√
*Vibrio cholerae* J3324-II	1.0 × 10^−4^	10.990 ± 0.24	0.261 ± 0.02	18.299	100.00	√
*Vibrio cholerae* J3330-I	1.0 × 10^−4^	2.800 ± 0.43	0.261 ± 0.02	7.926	25.48	√
*Vibrio cholerae* J3330-II	1.0 × 10^−4^	1.864 ± 0.34	0.261 ± 0.02	5.128	16.96	√
*Vibrio cholerae* CDHI5294-II	1.0 × 10^−4^	3.705 ± 0.41	0.261 ± 0.02	8.124	33.71	√
*Vibrio cholerae* UVC1324	1.0 × 10^−4^	10.904 ± 0.25	0.261 ± 0.02	18.115	99.00	√

**√-**DPV peak current is significantly higher than the control’s response. **× −** DPV peak current is significantly lower than the control’s response at 95% confidence level and 4 degrees of freedom

Recovery of *V. cholerae* J3324 and *V. cholerae* UVC1324 DNAs at three different concentrations spiked into the hybridisation buffer demonstrated 91.4 ± 2.2% to 108.9 ± 4.8% (*n* = 3) of recoveries percentage (Table 4). This result suggests that the proposed PSA-AuNPs-based electrochemical DNA biosensor could be adopted for highly reliable and accurate detection of *V. cholerae* DNA in environmental and clinical samples.

**Table 4:** Recovery of *V. cholerae* J3324 and *V. cholerae* UVC1324 DNAs by using the proposed PSA-AuNPs-based electrochemical DNA biosensor

Bacteria (μg/μL)	Current (μA), *n* = 3	Found (μg/μL)	Recovery (%)
*Vibrio cholerae* J3324			
1.0 × 10^−4^	12.82 ± 0.35	1.07 × 10^−4^	107.2
1.0 × 10^−5^	11.56 ± 0.22	9.14 × 10^−6^	91.4
1.0 × 10^−6^	10.41 ± 0.37	9.55 × 10^−7^	95.5
*Vibrio cholerae* UVC1324			
1.0 × 10^−4^	12.76 ± 0.56	9.57 × 10^−5^	95.7
1.0 × 10^−5^	11.61 ± 0.43	1.01 × 10^−6^	100.5
1.0 × 10^−6^	10.48 ± 0.48	1.09 × 10^−6^	108.9

### Performance Comparison with Other Reported *V. cholerae* DNA Biosensors

Based on the data summarised in Table 5, the proposed electrochemical DNA biosensor based on PSA-AuNPs immobilisation material shows an exceptional broad linear quantification range compared to other planar two-dimensional electrodes as the DNA supporters. This clearly demonstrates the advantage of the micro-sized latex particles where the polymeric PSA is capable to intensify the probe binding capacity with a simple loading method via the classical EDC/NHS coupling compared to avidin-biotin technology [24, 26] and ultra-low detection limit in zeptomolar range with reasonable assay time.

**Table 5:** A comparison of the developed DNA biosensor performance with other previously reported electrochemical DNA biosensor for the determination of pathogenic *V. cholere* DNA

Material and electrode design	Linear range (M)	LOD (M)	Hybridi-sation time (min)	Reference
DNA-PSA-AuNPs-carbon SPE (Direct immobilisation of capture probe on PSA gold-matrix)	1.0 × 10^−21^–1.0 × 10^−8^	1.0 × 10^−21^	60	Present work
AuNPs-PSA-Avidin conjugate label on reporter probe	1.0 × 10^−18^–1.0 × 10^−15^	1.0 × 10^−15^	30	Liew et al. (2015) [24]
AuNP-latex label on DNA reporter probe (Avidin-biotin binding)	1.0 × 10^−15^–1.0 × 10^−12^	1.0 × 10^−16^	-	Kuan at el. (2013) [30]
AuNP-latex label on DNA reporter probe (Avidin-biotin binding)	1.0 × 10^−12^–1.0 × 10^−9^	0.5 × 10^−16^	20	Pinijsuwan et al. (2008) [29]
AuNP-latex label on DNA target (streptavidin-latex/biotin-AuNP)	1.0 × 10^−12^–1.0 × 10^−9^	1.0 × 10^−12^	20	Kawde & Wang (2003) [35]

## Conclusions

This study reports the development of an electrochemical DNA biosensor for the detection of one of the most devastating high-risk *V. cholerae* pathogens. The PSA-AuNPs-modified DNA biosensor can be used for direct detection of DNA of interest from the extracted DNA without the need of amplification reaction via conventional PCR method, which is commonly used in those previously reported *V. cholerae* DNA biosensors. In addition, no further dilution of the extracted DNA is needed as the high-capacity AuNPs-doped latex microspheres-based DNA biosensor is highly sensitive for the quantitation of DNA at extremely low level in sub zeptomolar range. Therefore, the electrochemical DNA biosensor is greatly suitable as a surveillance and diagnostic tool to control the epidemic of the fatal intestinal infection.
